# Advances in Delivering Oxidative Modulators for Disease Therapy

**DOI:** 10.34133/2022/9897464

**Published:** 2022-09-21

**Authors:** Wei Yang, Hua Yue, Guihong Lu, Wenjing Wang, Yuan Deng, Guanghui Ma, Wei Wei

**Affiliations:** ^1^ State Key Laboratory of Biochemical Engineering, Institute of Process Engineering, Chinese Academy of Sciences, Beijing, China; ^2^ School of Chemical Engineering, University of Chinese Academy of Sciences, Beijing, China; ^3^ Department of Orthopedics, Fourth Medical Center, General Hospital of Chinese PLA, Beijing, China

## Abstract

Oxidation modulators regarding antioxidants and reactive oxygen species (ROS) inducers have been used for the treatment of many diseases. However, a systematic review that refers to delivery system for divergent modulation of oxidative level within the biomedical scope is lacking. To provide a comprehensive summarization and analysis, we review pilot designs for delivering the oxidative modulators and the main applications for inflammatory treatment and tumor therapy. On the one hand, the antioxidants based delivery system can be employed to downregulate ROS levels at inflammatory sites to treat inflammatory diseases (e.g., skin repair, bone-related diseases, organ dysfunction, and neurodegenerative diseases). On the other hand, the ROS inducers based delivery system can be employed to upregulate ROS levels at the tumor site to kill tumor cells (e.g., disrupt the endogenous oxidative balance and induce lethal levels of ROS). Besides the current designs of delivery systems for oxidative modulators and the main application cases, prospects for future research are also provided to identify intelligent strategies and inspire new concepts for delivering oxidative modulators.

## 1. Introduction

The oxidative level of cells is highly correlated to the physiological functions. Oxidative molecules are the products of aerobic metabolism of the body; the existing research has shown the oxygen molecules in appropriate doses can be used as a signaling molecule in the physical environment, which play an important signal regulating function [1]. For example, ROS is involved in cell division, migration, and contraction by influencing intracellular signaling pathways [2]. However, when cells undergo stressful conditions or diseases, the accumulated abnormal level of ROS leads to lipid peroxidation and DNA damage or even apoptosis [3]. In most cases, unexpected upregulation of ROS in normal cells causes cell dysfunction, inflammation, and various diseases, which should be avoided. However, an upregulated peroxidative state can be desired as a tumor elimination strategy, because such states damage the pristine antioxidant system or induce the lethal level of ROS in tumor cells [4]. Thus, oxidative modulators including antioxidants and ROS inducers are being exploited to achieve homeostasis maintenance or tumor cytotoxic effects [5]. Specially, supplementary exogenous antioxidants (e.g., vitamins, plant extracts, selenium, zinc, and inorganic nanoparticles with inherent antioxidant properties) can directly react with active oxygen or interrupt the oxidation reaction [6]. On the other aspect, ROS inducers refer to various agents (e.g., photosensitizers and sonosensitizers, liquid metals, and Fenton catalysts) designed to upregulate oxidative level to kill tumor cells [7], such as damaging the pristine antioxidant system to reduce ROS scavenging or inducing lethal levels of ROS by single therapy (e.g., photodynamic therapy) and combined therapy with chemotherapy or immune regulation [8].

**Figure 1:**
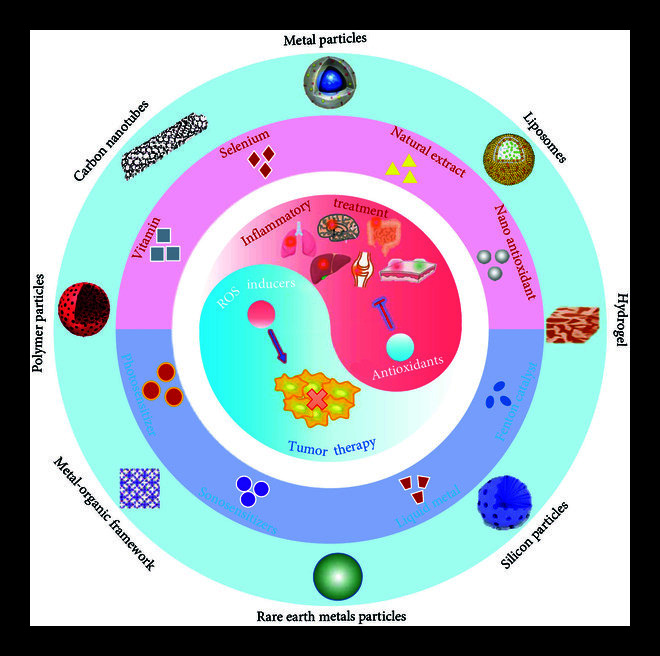
Delivery strategies that include divergent aspects of oxidative modulators.

Although oxidative modulators have attracted tremendous interest in recent years, their efficacy is unsatisfactory due to several problems such as poor stability, short blood circulation time, and poor targeting. In this sense, designing delivery vehicles to encapsulate oxidative modulators for targeted sites and improve bioavailability is highly demanded. Delivery vehicles (e.g., liposomes, polymer delivery systems, inorganic delivery systems, and other types of delivery systems) can be designed in a reasonable way according to the characteristics of the modulators [9]. At present, delivery systems for oxidative modulators involved in food or cosmetics are well documented or reviewed [10, 11]. However, a systematic review that refers to delivery systems for divergent oxidative modulators (both antioxidants and ROS inducers) within the biomedical scope is lacking. A comprehensive summarization and analysis of pilot research work is urgently needed to identify intelligent strategies and inspire new concepts for delivering oxidative modulators. To this aim, we review current delivery system designs for oxidative modulators (Figure 1), focus on the main application cases for inflammatory treatment (yin) and tumor therapy (yang), and provide prospects for future research.

## 2. Oxidative Modulators and Delivery Candidates

### 2.1. Oxidative System

The oxidation reaction occurs continuously, and the ROS generated in this process is of great significance to cell homeostasis. An appropriate level of ROS is beneficial for maintaining normal cell functions. To maintain the balance of ROS production and consumption, there is an antioxidant system inside the cell to scavenge excess ROS. The antioxidant system includes antioxidant molecules (e.g., uric acid, glutathione (GSH) and melatonin) and antioxidant enzymes (e.g., superoxide dismutase (SOD), catalase (CAT), and glutathione peroxidase (GSH-Pxs)) [12]. In detail, uric acid is the most concentrated of all blood antioxidants and is responsible for half of the total antioxidant capacity in human serum [13, 14]. GSH is a polypeptide containing cysteine and the sulfhydryl group on cysteine is reductive and can be reduced after oxidation [15]. SOD can catalyze the disproportionation and block the generation of free radicals [16]. CAT catalyzes the decomposition of hydrogen peroxide (H_2_O_2_) into water and oxygen in the cell, which prevents H_2_O_2_ from reacting with superoxide free radicals to generate hydroxyl free radicals, blocks the accumulation of hydroxyl radical (·OH), and ultimately reduces oxidative stress [17]. GSH-Pxs can effectively scavenge active free-radical molecules, prevent membrane lipid peroxidation, and scavenge H_2_O_2_. Under the catalysis of these enzymes, ROS exceeding the normal level can be scavenged in time to prevent accumulation [18].

Once the amount of high ROS in the cell exceeds the cell’s own oxidation scavenging ability, the antioxidant system will be unbalanced, resulting in the occurrence of oxidative stress. When the body is aging or in an abnormal state, an increased concentration of ROS in the cell can attack all the molecules due to its strong oxidizing ability. For example, active oxygen can cause lipid peroxidation of polyunsaturated fatty acids in biological membranes and initiate growth reactions to generate new free radicals, resulting in physiological disorders and pathological issues [19]. In addition, ROS can also react with nucleic acid bases to destroy bases and cause DNA damage [20]. In view of the ROS increase in the inflammatory sites, supplementing exogenous antioxidants is feasible to remove excess ROS and make up for the insufficient function of the pristine antioxidant enzymes. Considering the unique role of high levels of ROS in destroying cell structure and function, increasing the level of ROS will bring surprising results to kill cells such as tumor cells. In this sense, the regulation of ROS levels in the cells for different targets is of great significance.

### 2.2. Oxidative Modulators

To maintain the balance of ROS for inflammatory treatment or induce lethal level ROS for tumor therapy, a series of oxidative modulators have been designed for regulation of the oxidative level in cells. Among the modulators, antioxidants are the major types that reduce the active free-radical molecules in cells, which can protect cells from ROS damage. In contrast, ROS inducers are another modulator type that increases oxidative level or breaks the oxidative balance for tumor therapy.

#### 2.2.1. Antioxidants

Apart from endogenous antioxidants (e.g., SOD, CAT, and GSH-Pxs), additional supplementation with exogenous antioxidants is also an alternative method of maintaining the oxidative balance of the body [21]. Exogenous antioxidants can directly remove ROS or act as cofactors of enzymes to increase the removal of active free-radical molecules in the body. These antioxidants include organic sources (e.g., vitamin A, vitamin E, curcumin (CUR), quercetin (QU), resveratrol, and lycopene) and inorganic sources (e.g., selenium, zinc, and inorganic nanoparticles with inherent antioxidant properties) [6]. As shown in Table 1, clinical trials of antioxidants have focused on the treatments for inflammatory diseases or even the infectious disease COVID-19.

**Table 1:** Clinical studies related to antioxidants for disease treatment.

Antioxidant	Administration route	Phase	Diseases	NCT number	Ref
Vitamin C	Injection and oral	—	Wound healing in mandibular fracture	NCT03938584	—
Vitamin D	Dietary supplement	—	Innate immunity in elderly people	NCT03026244	[22]
Vitamin E	Oral	Phase 2	Airway inflammation	NCT03444298	—
Selenium	Injection	Phase 2	Neurological outcome after cardiac arrest	NCT01390506	—
Zinc	Oral	—	COVID-19	NCT04351490	[23]
Curcumin	Oral	—	Chronic kidney disease	NCT03475017	[24]
Quercetin	Oral	Phase 1	Chronic obstructive pulmonary disease	NCT03989271	[25]
		Phase 2			
Lycopene	Oral V8® low sodium	Recruiting	Metabolic syndrome	NCT03836651	—
Resveratrol and curcumin	Oral liposomed	Phase 2	Amyotrophic lateral sclerosis	NCT04654689	[26]

Among the organic antioxidants, vitamin E is a fat-soluble and widely used antioxidant that is also distributed on cell membranes [27]. It exerts antioxidant effect by inhibiting lipid peroxidation, which can protect the structural integrity and normal functions of biological membranes from oxidative stress. Similar to vitamin E, the main problems faced by antioxidants (e.g., CUR, QU, resveratrol, and lycopene) are the poor stability and hydrophobicity of the material extracts, and some are even sensitive to light and easily degraded [28, 29]. Therefore, it is necessary to improve the utilization of these antioxidants.

Among inorganic antioxidants, selenium is an element that exerts an antioxidant effect by maintaining the activity of glutathione peroxidase, which degrades hydroxide and maintains the stability of intracellular peroxide levels. Unlike selenium, zinc does not directly regulate the level of cell active molecules, but can bind to the sulfhydryl groups in proteins or compete with redox-active metals (iron/copper) in the membrane to protect the cell from oxidative stress damage. Moreover, some inorganic nanoparticles with inherent antioxidant properties (e.g., carbon-based nanoparticles [30], ceria nanoparticle [31], molybdenum-based nanoclusters [32], black phosphorus nanosheets [33], and DNA nanostructures [34, 35]) have been explored in biomedical applications for their ability to scavenge ROS. Most antioxidant particles are acting as ROS scavenger because of the susceptibility to be oxidized [32, 33], while specific DNA nanosheets react with ROS through the inherent DNA structure, thereby protecting normal tissues from attack [34, 35]. However, the physical and chemical properties of nanomaterials as well as their size, shape, and surface chemical functional groups, have important effects on antioxidant capacity *in vivo* and clearance in tissues and organs, which requires special attention.

#### 2.2.2. ROS Inducers

In eukaryotic cells, ROS are closely related to the occurrence and development of tumors, and increasing ROS to the lethal level has been shown to be conducive to tumor killing. To achieve this goal, several strategies have been developed. One strategy is to disrupt the endogenous oxidative balance, and the other is to increase ROS generation by delivering exogenous ROS inducers. Both strategies are expected to induce lethal levels of ROS and thereby kill tumor cells.

For strategies to disrupt endogenous oxidative balance, the corresponding ROS inducers are mitochondrial function blockers, antioxidant enzyme inhibitors, and antioxidant molecule depletion agents. (1) Mitochondrial function blockers are a class of agents that can disrupt the normal homeostasis of mitochondria and induce a large amount of ROS release, since 90% of ROS comes from mitochondria. These blockers mainly induce mitochondrial depolarization, disruption of the electron transport chain (e.g., *α*-tocopheryl succinate), and calcium homeostasis imbalance (e.g., calcium preparations). (2) Antioxidant enzyme inhibitors such as SOD family inhibitor 2-methoxyestradiol can inhibit the activity of enzymes, reduce the ability of scavenging ROS, and achieve the accumulation of ROS. (3) Antioxidant molecule depleting agents such as GSH synthesis inhibitors (e.g., oleanolic acid, sorafenib, and buthionine sulfoximine) and GSH depleting agents that contain groups that could react with the sulfhydryl group of GSH can reduce the excess GSH in tumor cells, and thereby reduce the growth of tumor and metastasis.

For the ROS generation strategy, corresponding ROS inducers such as photosensitizers, sonosensitizers, liquid metals, and Fenton catalysts are often involved in the delivery agents (Table 2). (1) Photosensitizers (e.g., Chlorin e6 (Ce6), indocyanine green (ICG), merocyanine (MC540), graphitic carbon nitride (g-C_3_N_4_), and black phosphorus (BP)) can transfer energy to reactants such as O_2_ under light conditions to produce ROS [36]. Among these photosensitizers, Ce6 is a degradation product of natural chlorophyll, which has a strong photodynamic reaction ability, large absorption coefficient in the infrared region, and low toxicity, but poor water solubility. (2) Sonosensitizers, such as semiconductor ZnO, are good choice with unique energy level structures and good chemical stability [37]. With ultrasound, ZnO improves the sonodynamic therapy efficiency by suppressing the rapid combination of electrons and holes to increase the quantum yield of ROS. (3) Liquid metals refer to metals whose melting point is close to or below room temperature. Among the liquid metals, an important class is eutectic gallium-indium alloys, which can produce ROS under microwave radiation. (4) Fenton catalysts (e.g., iron ions, copper sulfide, Cu^I^, and Mn^2+^) can mediate the Fenton reaction to achieve redox reactions and promote the generation of ROS [38–40]. Iron ions are the most widely studied metal ions with Fenton activity and have recently been popularly researched.

**Table 2:** Summary of ROS inducers in the treatment of tumors.

Inducer type	Inducer component	ROS type	Delivery system	Ref
Photosensitizers	Ce6	^1^O_2_	Nanoshell of Fe_3_O_4_@P-NPO/PEG-Glc	[41]
			Magnetic gold nanoheterostructures	[42]
	Indocyanine green (ICG)	^1^O_2_	Hepatitis B core protein virus-like particle (HBc VLP)	[43]
			Lipidosome	[44, 45]
	Graphitic carbon nitride (g-C_3_N_4_)	^1^O_2_, ·OH	Cu^2+^ and graphitic carbon nitride nanosheets	[46]
	Black phosphorus	^1^O_2_	Organic framework (MOF)	[47, 48]
			MnO_2_-laden black phosphorus nanostructure	[49]

Sonosensitizers	Rose Bengal	^1^O_2_	Microbubbles	[50]
	Semiconductor ZnO	^1^O_2_, ·OH	Defect-rich gadolinium (Gd) nanobullet	[51]

Liquid metal	Eutectic gallium−indium alloy	·OH, ·O_2_	Mesoporous ZrO_2_ nanoparticles	[52]

Fenton catalyst	Iron ions	·OH	Amorphous iron nanoparticles (AFeNPs)	[53]
			Dendritic mesoporous silica NPs	[54]
	Copper sulfide	^1^O_2_, ·OH	Manganese dioxide nanoparticles	[55]
	Cu^I^	·OH	Cu_2_(OH)PO_4_ nanocrystals	[56]
	Mn^2+^	·OH	Manganese-doped calcium phosphate nanoparticles	[57]

### 2.3. Delivery Systems for Oxidative Modulators

Recent delivery systems for oxidative modulators mainly involve liposomes, polymer particles, carbon nanotubes, mesoporous silica particles, metal particles, metal nanoshells, rare earth metal particles, MOFs, and hydrogels. Oxidative modulators can be encapsulated in or adsorbed on the surface of these delivery systems. By introducing the target molecules in these delivery systems, oxidative modulators will play a role in specific sites such as tumor sites or cells. An ideal delivery system should be characterized by a high loading, easy targeting, biodegradability, and high biocompatibility [58]. Here, we described the most representative delivery systems for oxidative modulators, including liposomes, polymer delivery systems, inorganic particles, and other types of delivery systems.

#### 2.3.1. Liposomes

Liposomes have vesicular structures that are assembled by a phospholipid bilayer and cholesterol with the phospholipid layer of the hydrophilic lumen. Hydrophilic drugs can be loaded inside the delivery systems, and hydrophobic drug molecules can be adsorbed between the phospholipid bilayer. Due to the advantages of high biocompatibility, strong drug carrying ability, and simple composition, liposomes have been approved by the Food and Drug Administration (FDA) [59]. For different types of oxidative modulators, liposomes can encapsulate both hydrophilic oxidative modulators (e.g., GSH) and hydrophobic oxidative modulators (e.g., vitamin E and curcumin) in the hydrophilic lumen and hydrophobic layer. Improving the stability of liposome preparations and preventing leakage can effectively improve the utilization of drugs. In addition, the gradual oxidation of unsaturated fatty acids in phospholipids will cause some antioxidants to play their role in advance and reduce the effective utilization rate of antioxidants, which needs special attention [60].

#### 2.3.2. Polymer Delivery System

Polymer delivery systems usually have a variety of structures and properties, which are designed to adapt to the packaging needs of different types of drugs [61]. Polymer delivery systems include polymer nanoparticles, micelles, hydrogels, and microparticles. Multiple drugs can be loaded to the surface of polymer particles *via* electrostatic adsorption or covalent cross-linking or embedded inside them for the delivery of different drugs. Surface modification or composition adjustment of the polymer may increase its stability and achieve controlled drug release. Because of their diverse composition and modifiable abilities, polymer delivery systems are attractive carriers for antioxidant and ROS inducers. Nevertheless, the wide distribution of polymer particles especially in micro size may be one of the problems in the delivery process of oxidative modulators, which correlates to the irregular distribution of polymer particles in organisms. Adjusting the size of polymer particles to maintain a uniform and stable state is conducive for the targeted release of oxidative modulators to inflammation or tumor sites.

#### 2.3.3. Inorganic Delivery System

Inorganic delivery systems include mesoporous silicon, carbon nanotubes, metal and metallic oxide nanoparticles, and rare earth metal particles. These delivery systems show different sizes, structures, and shapes under different preparation conditions [62]. With the large specific surface area and the natural substrate of mesoporous inorganic particles, they usually have high loading efficiency and unique physical/chemical properties. These properties impart additional functionalities that can deliver oxidative modulators. Inorganic delivery systems can be used as a multifunctional platform, and the abundant surface groups that can be easily modified and utilized to achieve a wide range of delivery applications [63]. For example, the unique properties of mesoporous silicon, such as its high loading capability and chemical and physical stability, enable it to deliver a variety of antioxidants and small molecule photosensitizers. In addition, carbon-based materials are good candidates as delivery carriers due to the high specific surface area and *π*-*π* stacking interaction for drug loading [64]. However, their biodegradability and biocompatibility might be a concern prior to the clinical applications of carbon-based materials [65].

#### 2.3.4. Other Types of Delivery System

Other types of delivery systems (e.g., emulsion and MOFs) have also shown the ability to deliver oxidative modulators. To our knowledge, studies have encapsulate different antioxidants using emulsions, such as conventional oil-in-water (O/W) emulsions [66], Pickering emulsions [67], and nano emulsions [68]. The characteristics of the emulsion are critical to the delivery of oxidation regulators, among which the loading efficiency, emulsion droplets size, and stability need to be considered [66]. Another type of delivery system MOF is an organic-inorganic hybrid material with intramolecular pores formed by self-assembly of organic ligands and inorganic metal ions or metal ion clusters through coordination bonds [69]. By choosing different organic ligands and metal ions, or changing the synthesis strategy, the size and shape of the pores of MOFs can be adjusted to meet the delivery requirements of divergent oxidative modulators [70, 71].

## 3. Delivery of Antioxidants for the Treatment of Inflammatory Diseases

Many studies have shown that the relationship between inflammation and oxidative stress is interdependent. On the one hand, inflammatory cells produce a large amount of ROS to kill invading pathogens during the process of inflammation. However, large amounts of ROS production may spread and lead to local oxidative stress. On the other hand, oxidative stress can also induce inflammation by activating the transcription factor NF-*κ*B to produce a large amount of inflammatory factors [72]. Oxidative stress has been associated with a variety of inflammatory diseases, including diabetes, cardiovascular disease, organ damage, skin inflammation and wound healing, bone diseases, and neurodegenerative diseases. In this sense, alleviating intracellular oxidative stress by antioxidants is feasible for the treatment of inflammatory diseases. However, using antioxidants alone is associated with a short circulation time, poor water solubility, and chemical instability. To avoid these defects, multiple delivery systems are designed to encapsulate antioxidants and transport them to inflammatory sites. Herein, we present examples of antioxidant delivery in the treatment of skin repair, bone-related diseases, organ dysfunction, and neurodegenerative diseases.

### 3.1. Antioxidant Delivery Systems for Skin Repair

Compared with other parts of the body, the skin is the outermost layer of the body, which is more directly affected by external stimuli and results in oxidative stress, causing a variety of skin diseases. For example, the skin is constantly exposed to oxidative conditions such as ultraviolet radiation, which can cause inflammatory erythema and aging of the skin. Moreover, the local trauma will be further aggravated with the inflammatory release of ROS, leading to refractory impaired wound healing. To treat these diseases, topical administration of antioxidants is of great benefit as they relieve oxidative stress and inflammatory response of cells, preventing the ROS diffusion into the systemic circulation with minimal systemic side effects by acting directly on pathological sites in the skin [73]. Nevertheless, the skin has a multilamellar sophisticated structure [74]. Conventional ointment is insufficient for topical treatment due to low penetration of drug molecules into the targeted skin tissues [75, 76]. In this case, a series of biomaterial systems for antioxidant delivery (hydrogels [77, 78], polymer membranes [79], and inorganic [80]/carbon [81]/metal [82] nanoparticles) with the functional adhesive coating (e.g., via chitosan or polydopamine) or skin-penetrating peptides [76] are emerging to overcome the skin barrier. Typical antioxidant delivery systems for treating skin inflammation, skin aging, and skin wound/healing (especially for diabetic wound healing) are introduced below.

For the treatment of skin inflammation, vitamins like vitamin A and vitamin E could act as free-radical scavengers against solar radiation, which are ideal drug candidates. However, the bioavailability and subsequent therapeutic efficacy are significantly compromised by the low water solubility of these vitamins. To solve this problem, Praca et al. constructed a water-in-oil microemulsion (ME) for encapsulating vitamin A and vitamin E [83]. The size of the ME was less than 200 nm, and the concentrations of vitamin A and vitamin E were 0.05% and 0.1% w/w, respectively. *In vivo* results showed that ME could significantly reduce ROS of inflammatory cells and the expression of INF-*α* compared with free vitamin, such a facile preparation method and the nanosize of ME, which not only facilitated the drug administration but also enhanced the drug permeation. This pilot construction of lotion encapsulated complex vitamins, providing a reference for the treatment of skin inflammation. Notably, when emulsions are used as delivery vehicles for antioxidants, the oil phase composition and saturation of the emulsion might affect the stability and even function of ME, which require rational selection and design to meet further delivery requirements.

Continuous UV exposure and ineffective ROS removal cause oxidative stress in the skin, resulting in damage to cell structures and tissue, and even the development of skin aging. To treat oxidative stress related skin aging (actinic keratosis), Campos et al. reported a liquid crystalline nanodispersion (LCN) loaded with lipoic acid (LA, an antioxidant that acts as the cofactor of mitochondrial enzyme) [84]. This LCN was composed of polar lipids and maintained a hexagonal phase structure even after drug, lipopolymers, and peptide additions, which are proved to be favorable for topical skin delivery. For a better efficacy, the LCN was also functionalized with cell-penetrating peptides (CPPs) (D4 peptide or TAT peptide), which could target the overexpressed epidermal growth factor receptor (EGFR) on the keratinocytes cell membrane. Once the TAT or DA functionalized LCNs were topically applied on porcine ear, the permeate amount of LA in the skin layer increased by 2.85-and 3.4-fold, respectively. The LA delivery system was demonstrated to prevent ROS damage, reducing the activity of myeloperoxidase (MPO) and the secretion levels of inflammatory cytokines (TNF-*α* and IL-1*β*).

**Figure 2:**
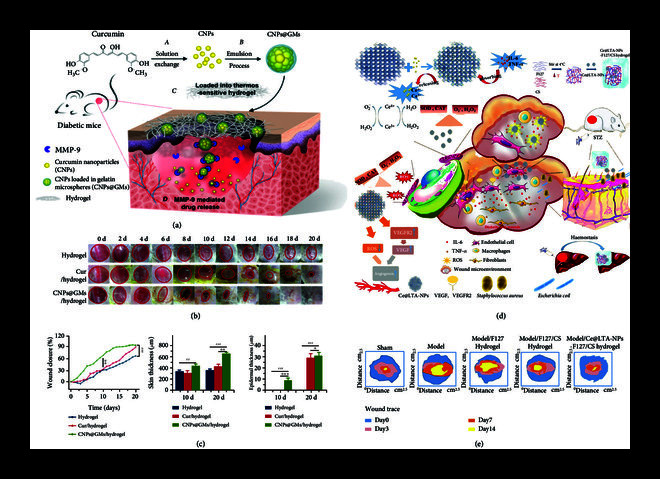
Schematic illustration of antioxidant delivery system for the treatment of diabetic wound healing. (a) Schematic representations of CNPs@GMs/hydrogel and the process of drug release at the wound bed in diabetic mice. (b) Representative images of wounds in diabetic mice for 0-20 days. (c) Dermal wound area (%), skin thickness, and epidermal thickness in the different groups. Reprinted with permission from Liu et al. [86] Copyright 2018, American Chemical Society. (d) Ce@LTA-NPs-F127/CS hydrogel was designed as a multi-target combination therapy for diabetic wound healing. (e) Traces of the wound closure during 14 days. Reprinted with permission from Qi et al. [77] Copyright 2021, Springer Nature.

For the wound healing, delivery systems that are ROS-sensitive that can enrich antioxidants or environmental responsible in wound tissue are desired. For example, Na et al. constructed ferrocene nanocapsules (FNCs) that can achieve the site-specific release of antioxidants (*α*-tocopherol, TP), since ferrocene could change from a hydrophobic state (at normal tissue) to a hydrophilic state at sites with high ROS levels [85]. The TP-loaded FNCs could effectively remove 40% of ROS, and the wound length was reduced from 139 *μ*m to 107 *μ*m with an accelerated healing speed (8 h). Skin wound healing in diabetic patients is a special refractory issue, which is usually associated with a disrupted balance between collagen degradation and the synthesis of extracellular matrix (e.g., MMP9 overexpression) upon high oxidative level. Inspired by this fundamental issue, Liu et al. constructed a thermosensitive hydrogel containing gelatin (MMP9 substrate) microspheres providing the antioxidant curcumin for diabetic wound healing (Figures 2(a)–2(c)) [86]. Curcumin nanoparticles were prepared by solvent exchange method and loaded into gelatin microspheres by emulsion process to obtain CNPs@GMs, which was mixed with thermosensitive hydrogel. GM degradation and size properties (4 *μ*m to 8 *μ*m) were tuned to achieve specific release of curcumin after degrading by MMP9 at the wound site. Through topical application in diabetic mouse model, CNPs@GMs could significantly promote wound healing, form granulation tissue, and increase skin/epidermal thickness. Besides the MMP overexpression, the bacterial infection is also a threat to the diabetic wound healing. To treat the refractory diabetes mellitus, Qi et al. prepared chitosan and Pluronic F127 thermoreversible hydrogel to encapsulate cerium doped in Linde type A (LTA) (Figures 2(d) and 2(e)) [77]. Cerium simulated SOD and CAT enzymes to eliminate ROS, while chitosan (CS) was used to resist bacterial infection at the wound site. In addition, the nanoparticles could neutralize inflammatory factors (IL-6) to remodel the wound microenvironment. This platform together showed a multistage therapy for diabetic wound healing with hemostatic and bactericidal behavior.

For the repairmen of skin inflammation, aging, and common/refractory wound, incorporating the functionalized delivery systems (e.g., emulsions, polymer/lipid particles, and hydrogels) with the antioxidants opens an era for effective topical management. To cope with the highly organized barrier as well as exert the drug efficacy, relative design concepts for delivery material/formulation/supplements focus on enhancing the penetration and enabling the site-specific or controlled drug release. Corresponding strategies refer to tuning the antioxidant solubility (via oil/water emulsion or flexible liposome), constructing reservoir in the skin (via flat carrier/gel or adhesive polymer), coupling the targeting peptides like CPPs, and integrating the merits of stimuli responding (for oxidative ROS, thermo, MMP overexpression) or enzyme simulation (for natural antioxidant). Moreover, with the development of novel materials and nanoparticles, a multi-scaled/targeted combination therapy is also feasible, which is especially favored for the refractory skin problem. It is also worth noting that when endowing delivery system with fascinating features the clinical value should be a critical point, as the existing dermal therapy is reasonably accepted for a long history.

### 3.2. Antioxidant Delivery Systems Used for Bone-Related Diseases

**Figure 3:**
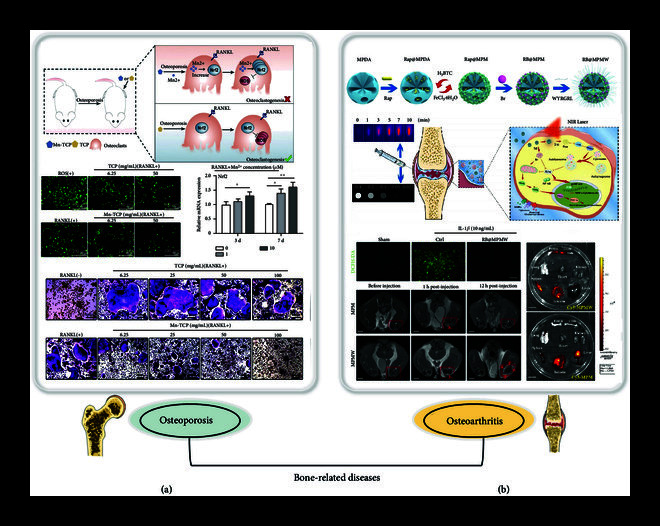
Schematic illustration of antioxidant delivery system for the treatment of bone-related diseases. (a) Schematic illustration of Mn-containing bioceramics inhibits osteoclastogenesis and promotes osteoporotic bone regeneration via scavenging ROS. Reprinted with permission from Li et al. [94] Copyright 2021, Elsevier. (b) Schematic illustration of cartilage-targeting peptide-modified antioxidant delivery nanoplatform for osteoarthritis therapy. Reprinted with permission from Xue et al. [96] Copyright 2021, Elsevier.

Bone-related diseases, such as osteoporosis [87, 88], osteoarthritis (OA) [89], rheumatoid arthritis [90], spinal degenerative disease [91], and femoral head necrosis [92], are common types of chronic diseases that cause inconvenience to the lives of patients. Among these diseases, osteoporosis, which often occurs in the elderly, has been widely studied due to the increasing aging of the global population and has been found to be associated with ROS in recent years. Excessive ROS accumulation may disrupt cellular homeostasis during bone remodeling. Scavenging excessive ROS can promote osteogenic differentiation and inhibit osteoclast differentiation, thus inhibiting bone mineral density and preventing osteoporosis. For example, Yu et al. developed nanoparticles (Fe_2_O_3_@PSC) to eliminate ROS to prevent osteoporosis with polyglucose-sorbitol-carboxymethyl ether (PSC) as the shell and Fe_2_O_3_ as the core [93]. After treatment with the nanoparticles, the ROS levels in MC3T3-E1 and RAW 264.7 cells recovered to normal levels, decreasing to 77.3% and 43.6%, respectively, by activation of the cellular antioxidant Nrf2/HO-1 pathway. Moreover, Fe_2_O_3_@PSC could promote osteogenic differentiation and inhibit osteoclast differentiation, and the number of osteoclasts was reduced by 36.6%. In another study, Li et al. prepared Mn-containing *β*-TCP bioceramics for the treatment of osteoporotic bone defects (Figure 3(a)) [94]. *β*-Tricalcium phosphate (*β*-TCP), a bioresorbable ceramic material, induces bone repair and regeneration. The addition of Mn-*β*-TCP to bone marrow mononuclear macrophages (BMMs) could significantly increase the scavenging of ROS such as free radical and superoxide radical and activate Nrf2 expression. After treated BMMs cells for 7 days, Mn-TCP inhibited the osteoclast differentiation of BMMs cells dose-dependently, which was demonstrated by TRAP staining images. Such a Mn-TCP bioceramic scavenged ROS by activating Nrf2, thereby inhibiting the generation of osteoclasts and promoting the regeneration of osteoporotic bone defects. This bioresorbable material was highly biocompatible and contributed to bone repair and regeneration, making it a potential delivery strategy.

Apart from osteoporosis, OA is another bone-related disease whose progression is closely related to oxidative stress, and scavenging excess ROS is an important treatment [95]. In the treatment of OA, systemic non-specific administration high-dose will reduce the therapeutic effect and increase systemic toxicity, and the use of targeted delivery system can avoid the above shortcomings. For example, Xue et al. designed a bilirubin (Br) delivery system based on MOF-decorated mesoporous polydopamine (MPDA) that can target chondrocytes (Figure 3(b)) [96]. The system consisted of rapamycin (Rap) loaded into the MOF mesopore and Br loaded into the MOF shell. Meanwhile, the collagen II targeting peptide (WYRGRL) was coupled on the surface of the above nanocarriers to form a cartilage-targeting double delivery nanoplatform (RB@MPMW). The results showed that the release of Br showed good free-radical scavenging and cartilage affinity. Meanwhile, due to the targeting effect of WYRGRL, RB@MPMW mostly targeted cartilage after injection, increasing the local concentration at the treatment site. Such a targeted delivery of antioxidants to cartilage provides an effective treatment strategy for bone and joint diseases. In addition, intraarticular injection is a feasible method for the treatment of osteoarthritis in order to maximize local effects. As injectable materials, hydrogel materials have been widely studied for their ability to achieve sustained drug release. For example, Cheng et al. reported a thermosensitive chitosan-gelatin-based hydrogel containing glutathione to evaluate the possible therapeutic effects of osteoarthritis [97]. The gel at 100 *μ*M showed interconnected porous structures that allowed for the free diffusion of GSH. The glutathione-loaded hydrogel of 100 *μ*M exists in liquid form at room temperature for a gel time within 69.5 s at body temperature. Compared with chitosan/GP hydrogels, the addition of gelatin increases the strength of hydrogels and reduces the gel time due to increased hydrophobicity, which potentially prevents rapid drug loss and may provide better sustained release performance. This injectable drug delivery system significantly reduced the expression of proinflammatory cytokines (TNF-*α*, IL-6, MMP-3, and MMP-9) and reduced cell apoptosis. This temperature-sensitive hydrogel material, which is liquid at room temperature, enables simple preparation of the material and is a potential therapeutic strategy as an injectable preparation.

Bone homeostasis is the balance between bone formation of osteoblasts and bone resorption of osteoclasts under fine biological control [98]. Elevated ROS induced by oxidative stress not only affects the process of osteoblast differentiation but also promotes osteoclast formation and inhibits osteoblast differentiation and growth by stimulating RANKL-induced osteoclast formation [99]. The following points need to be considered for the use of delivery systems to transport antioxidants for the treatment of bone-related diseases. First, the lack of site-specific delivery of antioxidants may increase osteocyte or even systemic toxicity and reduce therapeutic effectiveness. Therefore, the use of bone-targeting agents such as bisphosphonates and tetracycline, as well as osteoblast-targeting peptides and aptamer, to target specific cells in bone is highly desirable. Secondly, in terms of administration, local administration such as intra-articular injection in the treatment of articular bone diseases improves biosafety and enhances local effectiveness compared to systemic side effects caused by intravenous and oral administration. Thirdly, in terms of material selection, materials that can be absorbed and utilized by bone cells, such as bioceramics, calcium phosphate nanoparticles, and hydroxyapatite, are more conducive to inducing bone reconstruction. Although the use of delivery systems to treat bone disorders requires consideration of many aspects, the use of antioxidants has great clinical potential and even additional therapeutic effects compared to traditional drug therapy, which is commendable.

### 3.3. Antioxidant Delivery Systems Used for Organ Dysfunction

Organ dysfunction, such as liver dysfunction, kidney injury, lung injury, and inflammatory bowel disease (IBD), refers to abnormalities in organ function and even organ failure caused by a variety of pathogenic factors (e.g., infection and drug induction). For example, hepatic ischemia-reperfusion injury (IRI) is a kind of severe liver dysfunction associated with the production of large amounts of ROS [100]. The liver is highly regenerative; it can function properly with only 30 percent of it left. However, the production of a large number of ROS caused by acute infection will lead to lipid peroxidation and a large number of liver cell death and serious symptoms such as liver fibrosis, cirrhosis, and liver failure. To treat this disease, Long et al. reported the treatment of hepatic IRI with a hydrophilic carbohydrate-derived nanoparticle (C-NPs) as a nanoantioxidant. The antioxidant capacity of C-NPs is based on radical capture and reaction on conjugated *π*-systems and unsaturated bonds. Nanoparticles with an average diameter of 78 ± 11.3 nm derived from hydrophilic carbohydrates were synthesized by the one-step aqueous method [30]. *In vitro* antioxidant experiments showed that the activity of C-NPs was dose-dependent and could effectively scavenge O_2_^-·^and ·OH. Due to its strong ROS scavenging ability, the C-NPs inhibited the activation of macrophages, prevented the release of proinflammatory factors, and reduced the proliferation of neutrophils, thus effectively inhibiting the continuous occurrence of inflammation. Severe liver damage may cause functional failure, and increasing the local concentration of antioxidants in liver cells by targeting them can reduce ROS and alleviate liver damage. Zhao et al. used liver targeting of glycyrrhizin to encapsulate QU into nanogels to form gel particles (QU-GL) with a size less than 200 nm for the treatment of acute liver failure [101]. The antioxidant activity of quercetin in the nanogel was increased by 81-fold, which significantly reduced the dosage and toxicity of the antioxidant, and avoided the cytotoxicity observed with high doses of quercetin.

Damage to renal tubular pithelial cells is the key pathogenic factor of acute kidney injury, while oxidative stress is the key destructive factor of renal tubular epithelial cell injury [102]. Since the generation of ROS is mainly concentrated in mitochondria, it is of significance to target antioxidants to mitochondria of renal tubule epithelial cells and reduce mitochondria-specific oxidation outbreaks to reduce kidney injury. Wang et al. developed nanoparticles composed of triphenyl phosphine (TPP) modified low molecular weight chitosan-curcumin conjugate (TPP-LMWC-CUR, TLC), which have the ability to target mitochondria and have a good therapeutic effect on acute kidney injury *in vivo* [103]. With the good solubility of low molecular weight chitosan, the water solubility of conjugated CUR was improved, which was 288 times higher than that of free CUR. After treatment with TCL, the release of myeloperoxidase, SOD, GSH, and inflammatory factors (TNF-*α* and IL-6) decreased, and the survival rate of human renal proximal tubular cells (HK-2) was increased by 78%. In addition, since DNA molecules are sensitive to ROS, strategies using nucleic acid frameworks as ROS scavengers have emerged in recent years. Fan et al. demonstrated that rectangular DNA nanostructures (rDON) had kidney targeting and protective effects [35]. On this basis, ROS was scavenged in renal cells by constructing rectangular DNA nanostructure carriers with high kidney targeting to alleviate oxidative stress in acute kidney injury [34]. The results showed that the synthesized rDON had significant ability of ·OH scavenging, and significantly reduced the level of malondialdehyde (MDA) in renal homogenized cells during the oxidative stress accumulation of renal ischemia-reperfusion. Such new type of ROS scavenger, composed of programmable frame nucleic acid, can be reasonably designed according to different treatment sites and has broad application prospects.

**Figure 4:**
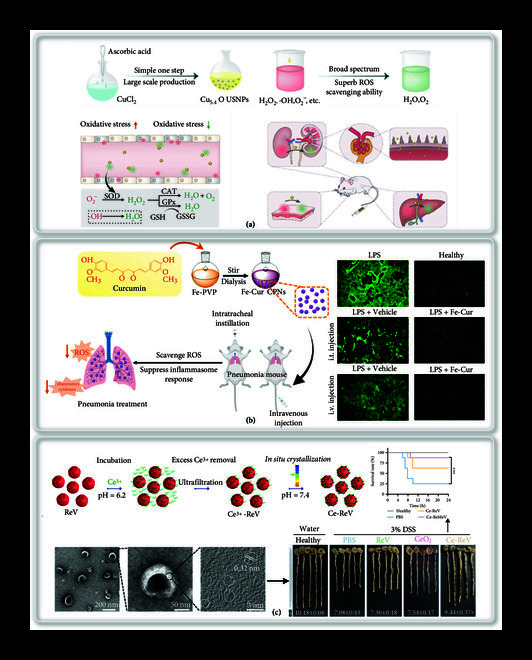
Schematic illustration of antioxidant delivery system for the treatment of organ dysfunction. (a) Schematic illustration of Cu_5.4_O ultra-small nanoparticles in the treatment of ROS-related diseases. Reprinted with permission from Liu et al. [105] Copyright 2020, Nature Publishing Group. (b) Schematic illustration of Fe-Cur NP synthesis and Fe-Cur NP based treatment for ALI in mice and fluorescence images of ROS stained lung slices. Reprinted with permission from Yuan et al. [106] Copyright 2021, American Chemical Society. (c) Schematic for the preparation of Ce-ReV. TEM images of Ce-ReV stained with uranyl acetate and high-resolution TEM image of cerium oxide nanocrystals on the ReV. The cerium oxide nanocrystals are indicated by white curves, and the corresponding lattice spacing is labeled by white line. Representative pictures of dissected colon tissues and total colon length. Survival rates of mice in each group monitored for 24 h. Survival rate was detected from each of 8 mice (n =8). Reprinted with permission from Zhao et al. [107] Copyright 2021, Elsevier.

In addition to inorganic nanoparticles and natural antioxidants, metal oxide nanoparticles have been reported to exhibit similar antioxidant enzyme activity to treat inflammation related to kidney injury [104]. For example, Liu et al. developed an ultra-small copper nanoparticles Cu_5.4_O NPs (Cu_5.4_O USNPs) by a simple one-step method to treat acute kidney injury (Figure 4(a)) [105]. Cu_5.4_O USNPs have similar functions as CAT, GPX, and SOD and thus can effectively remove ROS such as O_2_^-^·and ·OH and alleviate inflammation. Compared with other metal-based nanomaterials, the working concentration for ROS scavenging at the cellular level of Cu_5.4_O USNPs was only 25 ng·mL^-1^, which was nearly 2-3 orders of magnitude lower, thus indicating a higher ROS removal0 rate. Compared with natural enzymes, Cu_5.4_O USNPs had much higher thermal stability and pH stability and were easily recycled. The results showed that Cu_5.4_O USNPs could maintain the high expression of antioxidant genes by protecting cells from ROS damage, which might have a broad-spectrum effect on a variety of diseases related to oxidative stress. Besides the kidney injury, other inflammation diseases that were caused by uncontrolled inflammation can also be treated by metal nanoparticles *via* modulating excessive ROS. Yuan et al. reported that iron-curcumin-based nanoparticles (Fe-Cur NPs) with nanoenzyme function guide intracellular ROS clearance to treat acute lung injury (ALI) (Figure 4(b)) [106]. Lung slices showed that Fe-Cur NPs could effectively remove free radicals, and the lung tissues basically returned to normal. This work provides a prospect for curing ALI and is supposed to facilitate the patients to survive after pneumonia (like COVID-19) infection.

IBD is an intestinal inflammatory disease that seriously affects the quality of life of patients. Oral administration can directly act on the site of inflammation and inflamed colon lesions, and improve the patient’s compliance. For example, Zhao et al. reported a system that combines multienzyme mimic CeO_2_ nanoparticles with clinically approved montmorillonite (MMT), which is well tolerated by the digestive tract, for the treatment of inflammatory bowel disease [108]. These synthetic CeO_2_@MMT particles were less than 5 nm, which showed nanoenzymes property that mimic with both SOD- and CAT-like activities. CeO_2_@MMT eliminated 73.33% of intracellular ROS compared to cells treated with H_2_O_2_, which demonstrated the ability of the present system to eliminate ROS. In another study, Zhao et al. proposed to use erythrocyte vesicles as a carrier to deliver CeO_2_ to achieve a longer circulation time and large amount of accumulation in the acutely inflamed colon tissue after intravenous infusion (Figure 4(c)) [107]. Specifically, red blood cells were co-incubated with Ce^3+^ solution, and then cerium oxide nanocrystals were grown in situ on red blood cell vesicles (ReVs) membrane. Synthetic Ce-ReV could be observed under transmission electron microscopy, which could effectively remove free radicals in inflammatory parts, inhibited the decrease of colon length, and extended the life span. This one-pot synthesis of CeO_2_ nanoparticles based on cell vesicle templates was milder and more convenient than traditional hydrothermal methods and reduced the damage to vesicles. In addition, the system was upgraded by hybridizing ReV with mesenchymal stem cell-derived exosomes, showing additional repair functions for highly damaged tissues. The present work highlighted the incorporation of ROS clearance agents and the hybridization of cell membrane, which can be performed in a straightforward way with anti-inflammation efficacy and bring additional benefits owing to cell membrane.

For different organ injuries, the treatment modality using antioxidant delivery needs to be adjusted accordingly to meet the therapeutic needs of different organs. As with antioxidant delivery for renal disease, the size of the delivery carrier determines the ability to interact with the kidneys. In order to maintain accumulation in renal tissue without being filtered, the size of the carrier should be larger than the size of the glomerular filtration barrier (5-7 nm), but at the same time ensure permeability into the renal vasculature. For delivery systems used in pulmonary diseases, pulmonary inhalation of the delivery modality results in more than 3-fold higher distribution of the delivery particles in the lung compared to injection and oral administration. In addition, the size of the delivery vehicle has a direct impact on its site of deposition in the lung. Carriers with a size of 1-2 *μ*m are deposited into the bronchi, while nanoscale particles can be delivered to the lower respiratory system including the alveoli. In addition, oral administration is the preferred route for the treatment of intestinal diseases, but additional consideration needs to be given to the choice of materials to ensure that antioxidants are not damaged in the special environment of the digestive tract, such as a highly acidic environment. At present, it is a promising strategy to use probiotics as targeted drug delivery vectors with the continuous development of genetically engineered probiotics.

### 3.4. Antioxidant Delivery Systems Used for Neurodegenerative Diseases

The central nervous system is particularly susceptible to ROS due to its high metabolic rate and structural features, and neurodegenerative diseases are closely associated with redox homeostasis. Loss of mitochondrial function, altered metal homeostasis, and inactive oxidative defense mechanisms directly affect synaptic activity and neurotransmission of neurons, leading to cognitive dysfunction. Abnormal cellular metabolism affects the production and accumulation of amyloid beta (A*β*) and hyperphosphorylated Tau proteins, which in turn aggravate mitochondrial dysfunction and ROS production, leading to a vicious cycle. At present, various methods to prevent oxidative stress and improve neurological diseases have been proposed. Among them, the application of exogenous antioxidants is an effective strategy that has been gradually accepted. In this strategy, Song et al. have comprehensively summarized the antioxidant strategies for neurodegenerative diseases [109]. Herein, we illustrate the use of an antioxidant delivery system in the treatment of neurodegenerative diseases, especially Parkinson’s disease (PD) and Alzheimer’s disease (AD).

PD is a common neurodegenerative disease of the elderly, and it is characterized by tremors, limb muscle stiffness, slowness of movement, and disordered balance. Parkinson’s disease is a degenerative condition of nerve cells that produce the neurotransmitter dopamine (DA), resulting in insufficient dopamine production. At present, dopamine supplementation is one of the common treatments for this disease. However, because the central nervous system is protected by the blood-brain barrier (BBB), DA, a large molecule drug, has difficulty penetrating the BBB and performing their functions. Single-armed carbon nanotubes (SWCNTs) have variable structures, are able to penetrate cell membranes, and have high drug loading capacity, making them a potential delivery system that can penetrate the BBB. Guo et al. developed functionalized SWCNTs for the delivery of DA to the brain to treat neurodegenerative disease [110]. The surface of SWCNTs was modified with PEG to increase the biocompatibility and half-life in the carrier, and lactoferrin (Lf) acted as a targeting ligand to facilitate targeted delivery of nanotubes to the brain. The length of SWCNTs-DA was less than 200 nm, which facilitated penetration of the BBB. The results showed that when the DA content was 25 mg·kg^-1^, the ROS level and the expression of the inflammatory cytokines IL-1 and TNF-*α* were significantly decreased by the CNT delivery system in mice. Hydrophobic carbon nanotube materials need to be grafted onto hydrophilic groups to increase solubility during delivery. Considering the accumulation of non-degraded CNT in the brain, the biosafety and biocompatibility of delivery materials should be comprehensively demonstrated.

Mitochondrial dysfunction caused by A*β* peptides is one of the main pathological indicators of Alzheimer’s disease, which has been considered a possible cause of AD. Mitochondrial dysfunction precedes the appearance of A*β* peptides, therefore protecting mitochondria from oxidative stress can be used to prevent and treat AD. Because cerium oxide smaller than 5 nm has significant free-radical scavenging ability, Kwon et al. constructed triphenylphosphonium-conjugated ceria nanoparticles (TPP-ceria NPs) that localize to mitochondria and suppress neuronal death in a 5XFAD transgenic AD mouse [111]. Ceria nanoparticles were synthesized using a modified reverse micelle method, and the core diameter of the nanoparticles was 3 nm, which could penetrate mitochondria. Due to the small core size (3 nm), small fluid dynamics diameter (22 nm), good colloid stability, positive zeta potential (45 mV), and hydrophobicity of nanoparticles, these particles penetrated the BBB and achieved mitochondrial targeting of SHSY5Y neurons. In 5XFAD mice *in vivo*, the injection of TPP-Ceria NPs significantly restored neuronal activity and maintained the normal crista structure and morphology of mitochondria, suggesting the potential of prevention and early treatment of AD. However, when the antioxidant was used alone, it is not satisfied to eliminate the previous A*β* plaques, which may require combined strategy to fulfill a complement clearance. Overall, the report demonstrated a mitochondrial strategy for treating neuroinflammation, thus providing insights into the treatment of AD and other neurodegenerative diseases.

BBB, the blood-cerebrospinal fluid barrier, acts as a barrier between central nervous cells and the peripheral circulatory system, and plays an elaborate protective role in the central nervous system through the diffusion of a harmful substance and the promotion of nutrient transport. But these barriers are also a major challenge for delivering drugs to the nervous system to treat neurodegenerative diseases. Understanding the mechanisms by which the blood-brain barrier is crossed is important for improving the effects of antioxidants on the brain. Positively charged carriers, for example, are more likely to penetrate the BBB and enter brain tissue. Carrier surface modification of transporter and receptor binding molecules such as apolipoprotein E3 transferrin or lactoferrin can enhance the targeting of target cells. Currently, non-invasive brain drug delivery materials such as liposomes and polymer nanoparticles have been widely studied for their biocompatibility, low antigenicity, high degradation, and high patient compliance. However, the side effects of various nanoparticles on target cells and other organs, drug loading, and off-target drug interactions with non-specific receptors are problems and challenges in the treatment of neurodegenerative diseases using nanodelivery vectors. Future technologies combined with high technology such as implantable devices or store-delivered chips can bring new prospects and promise.

## 4. Delivery of ROS Inducers for Tumor Therapy

Studies have shown that cancer cells are in a state of oxidative stress. In order to adapt to this state, the antioxidant system in cancer cells is enhanced [112]. For example, the increased glutathione content in cancer cells neutralizes the excess ROS produced in the cells, thus allowing cancer cells to adapt to oxidative stress for a long time, and it is difficult to break their internal balance [113]. Even so, the high oxidative stress state of cancer cells compared with normal cells leads to a relatively reduced antioxidant system in cancer cells and a higher sensitivity to ROS than that in normal cells [114–116]. Therefore, ROS enhancement strategies can achieve the killing of tumor cells, while the damage to normal cells is low. In this sense, researchers have proposed a method of amplifying the level of oxidative stress to kill cancer cells. The mechanism is primarily based on magnifying oxidative stress to treat cancer, regardless of tumor type. The ROS inducers are involved in divergent strategies for reducing ROS clearance or increasing ROS generation. One strategy is to disrupt the balance of the oxidation system by damaging the pristine antioxidant system. The other strategy is to increase ROS production, which refers to dynamic therapies including photodynamic therapy (PDT), sonodynamic therapy (SDT), microwave hyperthermia (MDT), and chemodynamic therapy (CDT). With the assistance and development of nanotechnology, a series of delivery strategies based on ROS inducers have been developed for tumor therapy.

### 4.1. Delivery for Disrupting the Endogenous Oxidative Balance

**Figure 5:**
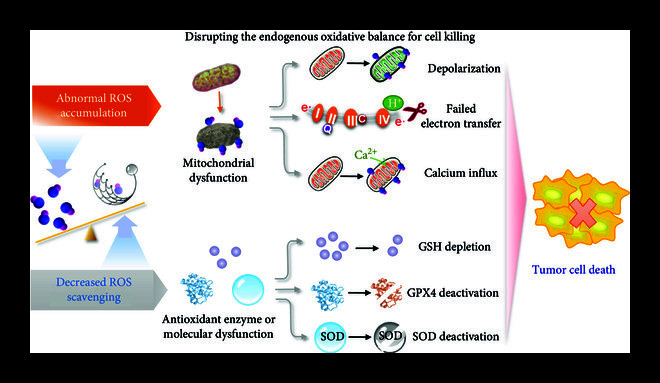
The antitumor delivery strategy for disrupting the endogenous oxidative balance of tumor cells.

The delivery to disrupt the endogenous oxidative balance is an important strategy for the treatment of cancer diseases, as an accumulation of ROS or decreased ROS scavenging occurred in the tumor cells during the process. As shown in Figure 5, these strategies include destruction of mitochondria to increase of mitochondrial ROS production and deactivation of endogenous antioxidant enzymes or molecules like GPX4, SOD, or GSH. In this sense, the imbalance of ROS generation/depletion system could induce excessive production of ROS, leading to subsequent death (e.g., apoptosis/ferroptosis) of tumor cells.

Since 90% of ROS in cells are derived from mitochondria, the mitochondrial dysfunction (e.g., loss of membrane potential, the compromised electron transfer chain or abnormal calcium influx) is an intelligent design concept for cancer treating. In particular, ROS are mostly produced in the mitochondrial electron transport chain, which can increase ROS production by disrupting mitochondrial respiration and breaking the balance of the electron transport chain. For example, Qu et al. designed a mesoporous silica particle with an average diameter of 68 nm targeting mitochondria *via* TPP, loaded with *α*-tocopherol succinate (*α*-TOS), which prevents ubiquinone from binding to succinate dehydrogenase (SDH), resulting in electron transport chain disruption and mitochondrial depolarization [117]. The results also demonstrated that the synthesized particles can reduce ATP synthesis and cause mitochondrial dysfunction. In addition, coenzyme Q10 is a key component of mitochondrial electron transport chain. Increasing the number of oxidative coenzyme Q10 and destroying the balance of the transport chain can increase ROS production. Dadali et al. reported a lipid-coupled non-nano-disperse BPM31510 of coenzyme Q10, which showed in clinical experiments that BPM31510 could reduce the oxygen consumption rate of pancreatic cancer, destroy its mitochondrial respiration, and induce the production of a large number of ROS to kill cancer cells [118].

Calcium overload is one of the most important means of inducing apoptosis. Calcium overload refers to the imbalance of calcium binding and free calcium homeostasis in mitochondria, which affects mitochondrial metabolism and ATP synthesis, leading to cell apoptosis. Zheng et al. co-mixed cisplatin, curcumin, and calcium carbonate into nanoparticles in a one-pot method, which could be released by contact in an acidic tumor microenvironment [119]. On the one hand, calcium carbonate can release a large number of calcium ions. On the other hand, curcumin can promote the release of calcium ions from endoplasmic reticulum to cytoplasm, inhibit the efflux of mitochondrial calcium ions, and further increase the calcium ion content in mitochondria, induce mitochondrial dysfunction, and thus induce apoptosis of cancer cells. In addition, acidification of the intracellular environment of tumor cells can cause Ca^2+^ influx leading to hypercalcium overload. Bao et al. loaded folic acid, upconversion particles, and photogenic acid molecules into MOF by one-pot method to form FMUP particles [120]. Under near-infrared light, FUMP can acidify tumor intracellular environment and release Fe^2+^. On the one hand, it can produce more ROS through the Fenton reaction. Meanwhile, an intracellular acidic environment can cause Ca^2+^ influx, which can effectively kill tumor cells in coordination with the Fenton reaction and Ca^2+^ overload.

Reducing ROS scavenging by disrupting the antioxidant system is another effective way to increase ROS accumulation in tumor cells. The antioxidant system can be destroyed by removing antioxidant molecules or destroying the activity of antioxidant enzymes. For example, GSH depletion is a potential strategy for eliminating antioxidant molecules. The high metabolic rate of tumor cells results in a high oxidation state. In response to this high oxidation state, tumor cells usually exhibit higher GSH levels than normal cells [121]. Destroying the original antioxidant system of tumor cells by increasing the consumption or blocking the synthesis of GSH will lead to an imbalance in the oxidative level of tumor cells. Among various nanoparticles, manganese-doped silica nanoparticles can be used as detection probes for GSH because the manganese-oxygen bond (-Mn-O-) in nanoparticles is easily reduced by GSH. In addition, sorafenib (SFB), which is an xCT inhibitor that decreases GSH production and suppresses the scavenging of lipid hydroperoxides, can inhibit the synthesis of GSH as a clinically approved drug. In this case, Tang et al. developed manganese-doped silica nanoparticles located in SFB (FaPEG-MnMSN@SFB) for the consumption of GSH to treat tumors [122]. In detail, manganese-doped silica nanoparticles (MnMSN) were synthesized by the one-pan method. SFB was loaded onto nanoparticles, and folate-grafted PEG (FaPEG) was modified on the surface by silica hydrolysis reaction to prolong the cycle time of nanoparticles. After HepG2 cells were incubated with FaPEG-MnMSN for 24 hours, the reduction in glutathione was 91.8%. In addition, this study showed that GSH clearance greatly prolonged the cell proliferation cycle and was conducive to inhibiting the proliferation of tumor cells.

Apart from depleting the antioxidant molecules to reduce ROS scavenging, damaging the activity of antioxidant enzymes is another method of disrupting the pristine oxidative system. Mitochondrial superoxide dismutase (SOD2) plays an important role in the elimination of mitochondrial ROS, and inhibition of SOD2 activity may lead to oxidative imbalance, increase intracellular ROS, and lead to tumor cell death. Zhang et al. reported a mitochondria-targeted mesoporous silica nanocarrier (2-ME/FA-Fe_3_O_4_@MSN) using 2-methoxyestradiol (2-ME, an inhibitor of the SOD family) and folic acids (FA) [123]. The accurate killing of HeLa cells *via* 2-ME/FA-Fe_3_O_4_@MSN was achieved through passive, active targeting (FA modifications and mitochondrial locating signals) and magnetic targeting (using an external magnetic field to direct the vector to the tumor cells). Compared with the same dose of free 2-ME, the production of mitochondrial ROS increased by 32%, and the number of HeLa cells undergoing apoptosis increased by 295%. As a new type of cell death, ferroptosis has become a potential target in cancer treatment. Ferroptosis requires accumulation of ROS and is manifested by accumulation of lipid peroxidation and reduction of glutathione peroxidase 4 (GPX4) [124, 125]. In this sense, destroying the enzymatic activity of GPX4 is a promising manner to amplify ferroptosis in tumor cells. Yu et al. reported a new type of metal-organic framework activated by the tumor microenvironment (TME) to induce GPX4 inactivation [126]. Specifically, this MOF is composed of Fe and Cu ions bridged by pegylated (FCSP MOF) disulfide bridges, which can be specifically degraded under redox TME. Depletion of GSH and inactivation of GPX4 synergistically lead to ferroptosis of tumor cells. This non-apoptotic nature makes ferroptosis-based cancer therapy a potential strategy for treating tumors and provides a target and theoretical basis for the development of new anticancer drugs and therapies.

### 4.2. Delivery of Photosensitizer for PDT

**Figure 6:**
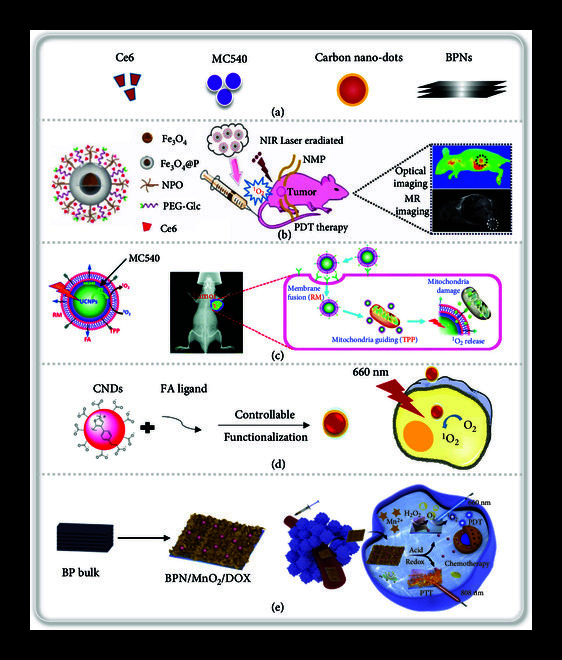
Representative ROS inducers and relative delivery applications for tumor therapy. (a) Representative photosensitizer of ROS inducers. (b) Formation of Fe_3_O_4_@P-NPO/PEG-Glc@Ce6 nanoprobes and photodynamic therapy in MGC-803 tumor-bearing mice. Reprinted with permission from Yin et al. [41] Copyright 2017, Nature Publishing Group. (c) Schematic illustration of UCNP-based nanoparticles loaded MC540 (PDT agents) on the basis of the PDT strategy. Reprinted with permission from Ding et al. [129] Copyright 2015, The Royal Society of Chemistry. (d) Schematic illustration of the covalent functionalization of CNDs and their targeted intracellular production of ROS. Reprinted with permission from Ji et al. [130] Copyright 2020, The Royal Society of Chemistry. (e) Schematic illustration of the preparation of multifunctional BPN/MnO_2_/DOX nanostructures and the systemic delivery of BPN/MnO_2_/DOX as a versatile theranostic platform for MRI-guided multifunctional therapy. Reprinted with permission from Wu et al. [49] Copyright 2019, Elsevier.

PDT has gradually developed into an effective method for the treatment of tumors. In PDT, photosensitizer at the tumor sites is stimulated by irradiation with excitation light of a specific wavelength, and the energy is transferred to the surrounding O_2_ to generate ROS to kill tumor cells [127]. At present, various photosensitizers have been developed, such as Ce6, MC540, carbon nanodots, and BPNs (Figure 6(a)). Ce6 is a photosensitizer of pigments with a small molecular weight that can be easily absorbed by tumor cells. However, its poor water solubility and easy metabolism hinder its accumulation at tumor sites. Yin et al. developed a ultra-small nanoplatform (Fe_3_O_4_@P-NPO/PEG-Glc@Ce6) that loaded Ce6, which effectively enhanced the accumulation of Ce6 in tumor [41]. The nanoplatform was composed of Fe_3_O_4_ as the core, NPO dendrimer, and PEGylated glucose (Glc) were loaded on the core, and Ce6 was loaded into the gap between PEGylated glucose and NPO dendrimer (Figure 6(b)). The nanoplatform had high stability and hardly changed after one month. Due to the ultra-small size and positioning effect of nanoplatform on tumors, the ingestion of the nanoplatform by tumor cells was higher than that of free Ce6. The average value of the fluorescence intensity was approximately 228-fold higher compared with free Ce6 after successive incubation of nanoprobe with MGC-803 cells. Under 633 nm laser irradiation, the tumor inhibition rate of the nanoprobe increased from 39% of free Ce6 to 77%. Ce6 was shielded by the NPO dendrimer, which maintained the monomeric form of Ce6, thus solving the problem of low photoreaction efficiency caused by the polymerization of the photosensitizer.

The choice of light wavelength in PDT therapy has a great influence on the tissue depth of treatment. Studies have shown that the penetration depth of near-infrared light with long wave is higher than that of UV-visible light with short wavelength. However, most traditional photosensitizers are excited by short wave, so the tissue penetration ability of PDT is limited [128]. Upconversion material UCNP can convert near-infrared light to short-wavelength UV-visible light for excitation of photosensitizer, so as to overcome the tissue penetration limitation of excitation light source. For example, Ding et al. developed upconversion nanoparticle (UCPN) based erythrocyte membrane (RM) coated biomimetic photodynamic therapy agents, which increased the circulation time of nanoparticles in the body due to its good biocompatibility. The nanoparticles were designed to target the mitochondria through TPP, and a large amount of singlet oxygen was produced to enhance the photodynamic therapy [129]. Specifically, the photosensitizer MC540 was incorporated around the core of UCNPs, and FA-targeted tumor cells and TPP-targeted mitochondria were modified on the surface (Figure 6(c)). The size of the particles was 45 nm, which had good stability and could fulfill the requirement for intravenous injection. Mitochondrial CLSM images showed that F/P-RM:Us/PS had a good targeting effect on mitochondria, and the targeting rate was as high as 90%. Under near-infrared irradiation, UCNP converted infrared light to visible light to activate the photosensitizer MC540 to generate singlet oxygen and directly acted on the cellular dynamic organs and effectively killed tumors. Compared with conventional PDT, this nanobiomimetic strategy uses cell membranes to evade capture by reticulo-endothelial system (RES) achieved more accurate targeting of cancer cells and mitochondria to increase cytotoxicity.

In addition to small molecule photosensitizers that produce ROS directly under light, the unique photophysical properties of carbon materials contribute to the light response, which is conducive to the production of ROS. For example, Ji et al. synthesized a red-emissive carbon nanodots (CNDs) and target tumor cells through FA ligand modification (Figure 6(d)) [130]. These carbon nanodots could be used for producing ^1^O_2_ in cells under laser irradiation, leading to the efficient death of cancer cells. In spite of the carbon nanodots, graphitic carbon nitride (g-C_3_N_4_) can also act as photosensitizer and exhibit catalytic activity due to its proper band gap and high specific surface area. Ju et al. reported a strategy for the integration of Cu^2+^ and g-C_3_N_4_ nanosheets to enhance the generation of ROS [46]. The photosensitizer g-C_3_N_4_ would generate ^1^O_2_ under light irradiation. Meanwhile, the metal reduction effect of copper catalyzed the reduction of molecular oxygen or H_2_O_2_ to generate ·OH. In this sense, improved ROS production was induced, and the viability of HeLa cancer cells was largely compromised.

Black phosphorus (BP) has the capability to produce ^1^O_2_ and can be used as a photosensitizer for photodynamic therapy. Wu et al. reported a class of compact MnO_2_-laden black phosphorus nanosponge (BPN/MnO_2_) (Figure 6(e)) [49]. This nanosponge increased the PDT of black phosphorus nanosheets by 3.8 fold compared with bare BP nanosheets through the inherent remarkable hypoxia amelioration. Since PDT relies on oxygen level, and the hypoxic tumor cells severely limit its effect, the oxygen complements strategy is explored to improve the PDT efficacy. Liu et al. loaded BQ (black phosphorus quantum dot) and CAT on the inner and outer layers of MOF by an in-situ growth method to form a heterogeneous structure of BQ-MIL@cat-MIL [47]. CAT catalyzed the generation of oxygen from H_2_O_2_ in the cell and delivered oxygen to the inner layer to relive the hypoxia issue. The BQ of the inner layer was irradiated by a laser and caused a large amount of singlet oxygen, thereby killing tumor cells. Due to a thickness-dependent band gap of bulk BP under visible photoirradiation, the ultrathin/small BQ is considered to be more acceptable photosensitizers for PDT.

### 4.3. Delivery of Sonosensitizers for SDT

In addition to laser radiation-mediated photosensitizers to generate ROS strategies, researchers found that sound can also induce sonosensitizers to produce ROS, which gave birth to SDT. SDT has great potential for application due to its high tissue penetration that uses a combination of ultrasound and sonosensitizer, which is more suitable for clinical transformation. There are two main types of sonosensitizers including organic sonosensitizers (e.g., hematoporphyrin monomethyl ether, rose bengal, and Dox) [131] and inorganic sonosensitizers (e.g., ZnO, TiO_2_, and carbon nanomaterials) [132]. Herein, we will introduce the strategies of SDT for the treatment of tumors, which are represented by the organic sonosensitizer Rose Bengal and the inorganic sonosensitizer ZnO.

As a diagnostic agent for ocular surface defects approved by the FDA, rose bengal is also a typical sonosensitizer with high biological safety. Hou et al. developed Rose Bengal microbubbles (RB-MBs) to increase the delivery efficiency of sonosensitizers, and the loading content of Rose Bengal was as high as 6.8% [50]. Upon ultrasound exposure, RB-MBs gradually collapsed into RB-NPs, resulting in the intracellular accumulation of RB being 7.5 times that of the group of RB-MBs without ultrasound treatment. In the HT-29 tumor model, the tumor suppression rate of RB-MBs+US (76.5%) was significantly higher than that of conventional MBs+US (commercial MB plus ultrasound, 23.8%) and RB-NPs+US (RB nanoparticles plus ultrasound, 49.2%). In addition, such microbubbles have excellent contrast enhancement ability in ultrasound imaging, which is very suitable for detecting the position and size of tumors.

Compared with organic sonosensitizers, inorganic sonosensitizers have the advantages of a unique energy level structure and good chemical stability. However, the low quantum yield of nano-sonosensitizers is the main problem faced by inorganic sonosensitizers (e.g., ZnO). Liu et al. used defect engineering to modify ZnO, which provides electron traps to inhibit the recombination of electrons and holes to increase the quantum yield, thereby enhancing the efficiency of sonosensitizers to produce ROS [51]. The defect-rich gadolinium-doped zinc oxide nanoprojectiles (D-ZnOx:Gd) prepared by the inert gas deoxidation method had an average hydrodynamic diameter of 187 ± 5.6 nm, which had a good passive targeting effect for tumor sites. At the cellular level, O_2_^-·^and ·OH were produced in 4T1 cells after D-ZnOx:Gd treatment after US irradiation, which significantly reduced the survival rate of 4T1 cells. *In vivo* experiments, intravenous administration of D-Znox:Gd resulted in loose tumor tissue and mass cell death, with less damage to normal cells and organs. It is worth noting that defect engineering can add more electrochemical energy storage sites and is widely used in battery materials. Existing metal oxides can be modified by defect engineering to provide electronic trap points and enhance the ROS generation efficiency to improve the performance of SDT.

### 4.4. Delivery of Microwave Sensitizer and Fenton Catalyst for Other Dynamic Therapies

MDT is widely used in the treatment of tumors because of the ability of microwave to penetrate deeply into tissues. Liquid metal is widely used due to its fluidity at room temperature and its metallic properties and can serve as a microwave sensitizer. Among them, eutectic gallium-indium (EGaIn) alloys can generate ROS under microwave irradiation and can be used for tumor treatment. Wu et al. reported a nanoparticle based on microwave radiation that could induce ROS and release heat at the tumor sites to kill cancer cells [52]. Specifically, the liquid metal and the ionic liquid were loaded into mesoporous ZrO_2_ nanoparticles, and the outer surface was modified with polyethylene glycol with good biocompatibility to construct PEG-IL-LM-ZrO_2_ nanoparticles. The prepared nanoparticles had a size of 210 ± 60 nm, and had certain stability under acidic and neutral conditions. Experimental results showed that the amount of ROS generated by nanoparticles under microwave irradiation was 3.7 times that of non-irradiation. The cell survival experiment showed that the ROS produced by microwave irradiation of nanoparticles had a lethal effect on cells. Since ZrO_2_ is a contrast agent, this kind of nanoparticle can also be used to monitor the treatment status in real time through CT.

CDT is a strategy that produces ROS through chemical reactions. CDT uses Fenton or Fenton-like reactions to convert H_2_O_2_ into ·OH, which is one of the most toxic ROS [133]. Ferrocene and its derivatives are typical Fenton catalysts, which can directly catalyze H_2_O_2_ to produce ROS under acidic conditions and convert H_2_O_2_ to O_2_ under normal pH, providing excess H_2_O_2_ and alleviating oxygen deficiency to enhance CDT in tumor cells. For instance, Sun et al. designed a nanoparticle that was loaded with ferrocene, glucose oxidase, and metabolic inhibitors, which inhibited the energy metabolism of tumor cells, by generating excessive ROS for mitochondrial damage, and realized the killing of resistant cancer cells. This enhanced CDT strategy provides a reference for effective treatment of resistant cancer and other metabolic disorders [134]. In general, the Fenton reaction is generally mediated by Fe^2+^, and the reaction rate can be accelerated under acidic conditions. However, tumor acidosis characterized by extracellular acidity (pHe ≈6.5) and intracellular alkalinity (pHi ≈7.2) severely limits the application of CDT. To solve this problem, Chen et al. constructed nanoparticles based on carbonic anhydrase IX (CA IX) inhibitors to induce H^+^ accumulation in tumor cells [53]. Specifically, AFeNPs@CAI was synthesized from amorphous iron nanoparticles with protocatechuate acid and CA IX inhibitor (CAI) through amination, and the particle size was 7-10 nm. Under the action of CAI, the acidity in tumor cells was enhanced, and the release rate of Fe^2+^ was significantly increased, which could promote the rate of Fenton reaction. The results of *in vivo* tumor xenotransplantation experiments showed that AFeNPs@CAI could significantly inhibit tumor growth and metastasis. Different from other methods that directly act on cancer cells, this method based on pH changing to reconstruct tumor acidosis and enhance CDT provides a new treatment idea.

**Figure 7:**
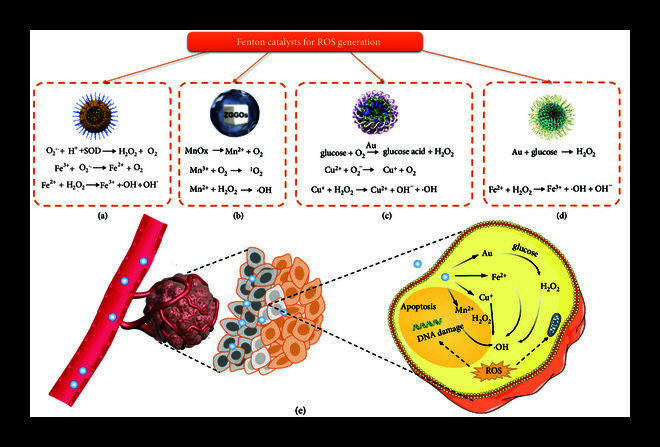
Schematic diagram of delivering Fenton catalysts for tumor CDT therapy. (a) Schematic diagrams of the structure of the OVA-NCs and reaction formula of ROS generation. Reprinted with permission from Jiang et al. [136] Copyright 2020, Wiley-VCH GmbH. (b) Schematic diagrams of Mn^3+^-rich oxide/persistent luminescence nanoparticles and reaction formula of ROS generation. Reprinted with permission from Ding et al. [137] Copyright 2021, Ivyspring International Publisher. (c) Schematic diagrams of hollow mesoporous organosilica biocatalysis nanoreactor and reaction formula of ROS generation. Reprinted with permission from Li et al. [138] Copyright 2020, WILEY-VCH Verlag GmbH & Co. KGaA, Weinheim. (d) Schematic illustration biomimetic inorganic nanoplatform and reaction formula of ROS generation. Reprinted with permission from Gao et al. [54] Copyright 2019, T WILEY-VCH Verlag GmbH & Co. KGaA, Weinheim. (e) Schematic diagrams of the nanodelivery systems passively enriched in tumor tissues by the EPR effect, and Fenton or Fenton-like reactions occur inside the cells to induce cell apoptosis.

In addition to Fe^2+^, other metal ions such as Fe^3+^, Cu^2+^, and Mn^2+^ can also catalyze Fenton-like reactions in tumors to produce ROS (Figures 7(a)–7(d)) [135]. Once the Fenton catalysts are transported to tumor cells by delivery vehicles, ·OH was generated through different reactions in tumor cells, causing DNA damage and mitochondrial destruction, and further leading to tumor cell apoptosis (Figure 7(e)). For example, Jiang et al. synthesized Fe_3_O_4_NPs-loaded albumin nanocapsules (OVA-NCs) as nanocarriers. The Fe^2+^-mediated Fenton reaction converted H_2_O_2_ into highly toxic ·OH. At the same time, oxygen was released when Fe^3+^ was converted to Fe^2+^, and the oxygen relieved tumor cell hypoxia (Figure 7(a)) [136]. Ding et al. reported chromium-doped zinc germanate (ZGGO) nanoparticles (Mn-ZGGOs) encapsulated by Mn^3+^-rich manganese oxide (MnOx) (Figure 7(b)) [137]. In the tumor area, the MnOx shell quickly decomposed to generate Mn^3+^ and oxygen (O_2_) to directly generate ^1^O_2_. The resulting Mn^2+^ converted endogenous H_2_O_2_ into ·OH through a Fenton-like reaction. Mn-ZGGOs also showed excellent T1-weighted magnetic resonance (MR) imaging and ultra-sensitive X-ray excited persistent luminescence (XEPL) imaging in tumors.

In addition to using Fenton catalyst alone, the combination with other synergists can meet the high demand for H_2_O_2_ of CDT on the basis of ensuring the efficiency of Fenton catalyst. For example, nanozyme such as Au nanoparticles can act as artificial enzyme (glucose oxidase) and efficiently catalyze glucose to generate H_2_O_2_. Li et al. reported an in-situ polymerized hollow mesoporous organosilicon nanoparticle (HMON) biocatalytic nanoreactor for enhancing ROS-mediated treatment of pancreatic duct adenocarcinoma (Figure 7(c)) [138]. Specifically, Au nanoparticles are fixed in HMON, and Tannic acid (TA) can be deposited on the surface of HMON. The digalloyl groups rich in TA can be used as a chelating site for Cu^2+^ to increase Cu^2+^ loading. Through the inorganic enzyme catalysis of Au nanoparticles and the Fenton-like catalyzed reaction of Cu^2+^, a large amount of highly toxic ·OH was produced in tumor cells, thereby causing cancer cells to undergo apoptosis. Similarly, Gao et al. reported a high-efficiency biomimetic dual inorganic nanoenzyme based on the ultra-small Au and Fe_3_O_4_ NPs that both loaded in mesoporous silica particles. Au NPs acted as glucose oxidative nanozymes to catalyze the oxidation of glucose to H_2_O_2_, and the generated H_2_O_2_ was subsequently catalyzed by peroxidase-mimicking Fe_3_O_4_ NPs to release ·OH in the acidic environment of the tumor. Such a catalytic reaction ensures efficient tumor killing (Figure 7(d)) [54].

### 4.5. Delivery of ROS Inducers for Combined Tumor Therapy

**Figure 8:**
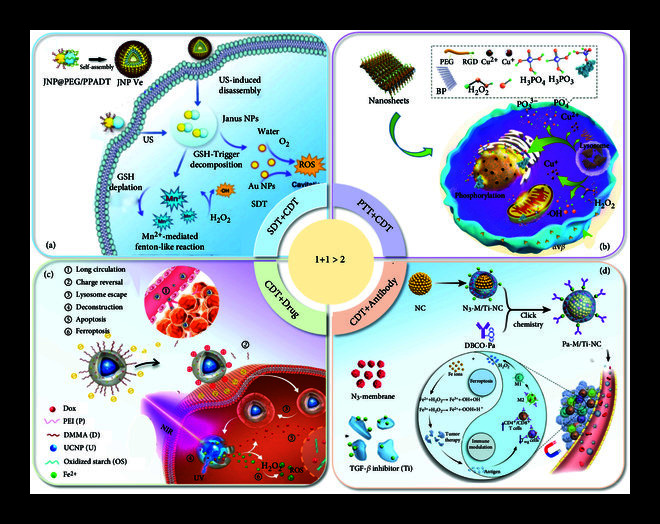
Schematic illustration of combined treatment strategy for delivering ROS inducers to tumor cells. (a) Schematic illustration of tumor treatment strategy combined with SDT and CDT. Reprinted with permission from Lin et al. [139] Copyright 2020, WILEY-VCH Verlag GmbH & Co. KGaA, Weinheim. (b) Schematic illustration of tumor treatment strategy combined with PTT and CDT. Reprinted with permission from Hu et al. [140] Copyright 2020, Nature Publishing Group. (c) Schematic illustration of nanolongan with multiple conversions and the corresponding anticancer mechanism. Reprinted with permission from Bao et al. [141] Copyright 2019, American Chemical Society. (d) Schematic illustration of a biomimetic magnetosome for ferroptosis/immunomodulation synergism in cancer. Reprinted with permission from Zhang et al. [142] Copyright 2019, American Chemical Society.

In view of the complexity and difficulty of tumor treatment, a combination of multiple tumor treatment strategies will strengthen the antitumor efficacy in comparison to single dynamic therapy. Lin et al. constructed a Janus nanoparticle, which combined SDT and CDT for more effective tumor therapy (Figure 8(a)) [139]. The cavitation effect of the sonosensitizer (Au NPs) enhanced the Fenton-like catalytic ability of Mn^2+^ and decomposed more H_2_O_2_ into ·OH. Such a synthetic efficacy is ascribed to the local turbulence of ultrasonic shock waves from SDT, which can promote the interaction between the catalysts and substrate therefore improve the effect of Fenton reaction. In addition, the combination of photothermal therapy (PTT) and CDT was also developed to increase the production of ·OH on the basis of local hyperthermia and improve the efficiency of tumor elimination. Hu et al. used BP nanosheets as a high-performance photothermal agent to realize the combination therapy through electrostatic attraction and coordination capture of Cu^2+^ (Figure 8(b)) [140]. Present types of combination therapy have greatly increased the efficiency of treatment and has produced gratifying results.

In addition to the above-mentioned two dynamic therapy combined or dynamic therapy combined with photothermal therapy (PTT), our team developed a combination of CDT and chemotherapeutic drugs to treat tumors with nanolongan (Figure 8(c)) [141]. Specifically, the upconversion nanoparticles and Dox were encapsulated in oxidized starch-based gel nanoparticles, which were then cross-linked with Fe^3+^ and modified with polyethylenimine (PEI) and 2, 3-dimethylmaleic anhydride (DMMA). DMMA offers a negatively charged surface, which prolongs the circulation time. The diameter of the nanolongan (DGU: Fe/Dox) was approximately 125.8 nm, and the weight percentages of iron and Dox were 11.84% and 10.07%, respectively, which were favorable for enhanced permeability and retention effects on surrounding tumors. In the slightly acidic environment of the tumor, the nanolongan was reversed to a positive charge due to the shedding of DMMA. This conversion promoted the escape of nanolongan from lysosomes. With near-infrared irradiation, the gel structure was deconstructed and Dox was dispersed into cells because Fe^3+^ transformed into Fe^2+^. At the same time, Fe^2+^ was released to react with H_2_O_2_ to produce a large amount of ROS. The results showed that this nanolongan was able to completely eliminate the tumor. The survival rate at 55 days was 100%, thus further demonstrating the effectiveness of the synergistic tumor treatment.

More and more attention has been paid to immunotherapy to activate the body’s immune cells to kill the tumor cells along with other therapy. In particular, elevated level of ROS (e.g., toxic ·OH) produced by Fenton or Fenton-like reactions leads to the mass death of immunogenic tumor cells. These tumor antigens released by the dead immunogenic tumor cells further activate the immune cells, thus achieving a strong combination of CDT and immunotherapy. For example, Zhang et al. developed magnetosomes (PA-M/Ti-NCs) for tumor therapy based on the synergistic effect of ferroptosis and immune regulation [142]. As shown in Figure 8(d), this magnetosome with Fe_3_O_4_ magnetic nanoclusters (NCs) was synthesized as the core prepared by a one-pot hydrothermal method, which provided abundant Fenton catalyst iron ions. Then, the NPs were coated with pre-engineered leukocyte membranes and TGF-*β* inhibitor (Ti). PD-1 antibody (Pa) was decorated on the magnetosome surface through mild and efficient click chemistry. The results showed that 100 *μ*g of NCs could be loaded with 20 *μ*g Ti, 50 *μ*g leukocyte membrane, and 10 *μ*g Pa. After injection, the NC core with magnetization and superparamagnetism enables magnetic targeting. Pa and Ti cooperate to create an immunogenic microenvironment, which increases the amount of H_2_O_2_ in polarized M1 macrophages, and the release of Fe particles promoted the occurrence of the Fenton reaction, thus inducing the ferroptosis of tumor cells and further regulating the tumor microenvironment. The results showed that Pa-M/Ti-NCs almost completely inhibited tumor growth compared to NCs alone. In seven different tumor cell models, PA-M/TI-NCs showed strong antitumor effects. This superior efficacy is closely related to the strategy of treating tumors by regulating the tumor microenvironment, which can significantly improve the prognosis of tumors. Regardless of the tumor types and antitumor therapy strategy, present combination modalities (CDT+SDT, PTT+CDT, CDT+Drug/Antibody) have achieved a “1+1>2”effect. Such a synergistic effect is indispensable of the intrinsic merit of the oxidative modulators and rational design of the delivery systems.

As tumor cells are more sensitive to oxidation state than normal cells, changes in oxidation level of tumor cells are caused by destruction of intracellular organelles, depletion of antioxidant enzymes/molecules, and delivery of ROS inducers, resulting in the increase of ROS and exceeding the threshold of tolerance value, resulting in the death of tumor cells. Even so, killing tumor cells by increasing ROS inevitably causes damage to oxidation-sensitive organs such as the liver, kidneys, and heart. In this sense, enhancing the availability of ROS inducers as well as reducing their potential toxicity to normal tissues is the major direction for developed delivery system. With arm of the intelligent delivery systems and combined modality, the ROS inducers can thus actively target tumor cells with neglect toxicity, achieving a win-win situation.

## 5. Summary and Outlook

The use of delivery systems to encapsulate oxidation modulators can avoid shortcomings (e.g., chemical instability and short blood circulation) and provide a new solution strategy for the treatment of various inflammatory diseases and cancers. On the one hand, lipid peroxidation and DNA damage can be reduced by downregulating ROS levels in inflammatory sites, thus treating inflammatory diseases (e.g., skin repair, bone-related diseases, organ dysfunction, and neurodegenerative diseases). On the other hand, increasing the ROS level in the tumor sites can kill tumors (e.g., disrupt the pristine oxidative system and induce lethal levels of ROS). In different disease states, oxidative stress should be downregulated or upregulated to maintain the health of the body.

Research on the oxidative stress system regulating various diseases has made great progress. However, challenges and additional problems still remain. First, an efficient delivery system should be rationally designed to maintain high loading and activity of the oxidation modulators, desired circulation time, and the spatio-temporal coupling release in the target cell. In coping with different oxidative regulators, specific physiochemical properties (e.g., size, shape, and structure) or divergent functions (targeting, loading, and shielding) could be fine-tuned or integrated into the delivery systems. Second, the application fields of oxidative modulators can be further expanded. Recent studies have shown that oxidative stress is one of the reasons for the aging of immune cells in the elderly. Therefore, by regulating the oxidative stress of immune cells, the function of immune cells in the elderly can be enhanced, thereby enhancing the response effect of the elderly to immunotherapy, which may be a new direction for future research. In addition, considering the tumor heterogeneity in ROS promotion strategies, specific design of ROS inducers against different tumor types is underdeveloped, and the efficacy discrepancy may be deduced for a more intelligent oxidative modulators delivery system. At the same time, the clinical translation and quality testing of oxidative modulators urgently need to be resolved. The industrial production of the delivery system, the development and optimization of the synthesis process, and the control of the production scale all require further research. With the in-depth research and resolution of these problems, additional tools can be provided for the delivery of oxidation modulators, and the clinical application of oxidative stress modification therapy in the treatment of various diseases is anticipated.
